# Label Propagation Prediction of Drug-Drug Interactions Based on Clinical Side Effects

**DOI:** 10.1038/srep12339

**Published:** 2015-07-21

**Authors:** Ping Zhang, Fei Wang, Jianying Hu, Robert Sorrentino

**Affiliations:** 1 Healthcare Analytics Research, IBM T. J. Watson Research Center, Yorktown Heights, USA; 2 Department of Computer Science and Engineering, University of Connecticut, Storrs, USA

## Abstract

Drug-drug interaction (DDI) is an important topic for public health, and thus attracts attention from both academia and industry. Here we hypothesize that clinical side effects (SEs) provide a human phenotypic profile and can be translated into the development of computational models for predicting adverse DDIs. We propose an integrative label propagation framework to predict DDIs by integrating SEs extracted from package inserts of prescription drugs, SEs extracted from FDA Adverse Event Reporting System, and chemical structures from PubChem. Experimental results based on hold-out validation demonstrated the effectiveness of the proposed algorithm. In addition, the new algorithm also ranked drug information sources based on their contributions to the prediction, thus not only confirming that SEs are important features for DDI prediction but also paving the way for building more reliable DDI prediction models by prioritizing multiple data sources. By applying the proposed algorithm to 1,626 small-molecule drugs which have one or more SE profiles, we obtained 145,068 predicted DDIs. The predicted DDIs will help clinicians to avoid hazardous drug interactions in their prescriptions and will aid pharmaceutical companies to design large-scale clinical trial by assessing potentially hazardous drug combinations. All data sets and predicted DDIs are available at http://astro.temple.edu/~tua87106/ddi.html.

Drug-drug interaction (DDI) may happen unexpectedly when more than one drugs are co-prescribed, causing serious side effects. DDI is a serious health and safety issue which draws great attention from both academic and industry^1^. As the number of approved drugs increases, the number of potential interactions between prescribed medications rapidly rises. Moreover, elderly patients and cancer patients are typically administrated numerous medications^2^,^3^, exposing them to a high risk of adverse DDIs. Discovering and predicting DDIs will not only prevent life-threatening consequence in clinical practice, but also prompt safe drug co-prescriptions for better treatments.

Most DDIs are discovered by accident in the clinic or during phase IV clinical trials that take place once a drug is already on the market^1^. In order to effectively detect DDIs, a number of statistical methods were developed for discovering DDIs from scientific literature^4^,^5^, electronic medical records^6^, insurance claim databases^7^, and the FDA Adverse Event Reporting System^8^,^9^. However, these methods still rely on the accumulation of sufficient clinical evidence in the post-marketing surveillance. As such, they are insufficient for detecting all DDIs and cannot alert the public to potentially dangerous DDIs before a drug enters the market.

In recent years, a new direction is to predict novel DDIs based on mechanistic and structural information of the drugs themselves and their interactions with proteins. For example, computational methods were developed for predicting DDIs by analyzing chemical structure similarity^10^, implementing the chemical-protein interactome^11^, modelling interaction profile fingerprints^12^, and exploiting pharmacointeraction network structure^13^. There are also some efforts on predicting DDIs by integrating multiple molecular and pharmacological data^14^,^15^. The advantage of these methods lies in the fact that they rely mainly on chemical and bioactivity data from laboratory studies rather than clinical records. As a result, they could potentially be used to predict DDIs years in advance, enabling drug safety professionals to better prioritize their limited investigative resources and take appropriate regulatory action.

Similarity-based approach is a representative strategy for DDI prediction^10^,^12^,^15^. However, most of the existing similarity-based DDI prediction algorithms only utilize first-order similarity (i.e., use immediate similarities for prediction) and doesn’t consider transitivity of similarity. To address this deficiency, we proposed a label propagation approach for predicting DDIs by considering high-order similarity. Furthermore, clinical phenotypic information has not been adequately investigated for its power in predicting DDIs. The advantages of leveraging clinical phenotypic information lies on two aspects: (1) clinical phenotypic information could serve as biomarkers for both therapeutic effects^16^,^17^ and toxic effect^18^, thus has potential to be used in DDI predictions. (2) Clinical phenotypic information is derived from direct observations from human, thus has better translational power when comparing with molecular or animal models^19^ in DDI predictions. In this study we also investigated the usage of clinical side effects (SEs) to predict DDIs.

To facilitate the use of different SEs and chemical structures information sources, we propose an integrative label propagation framework to predict DDIs by integrating SEs extracted from package inserts of prescription drugs, SEs extracted from FDA Adverse Event Reporting System, and chemical structures from PubChem. The proposed framework is also extensible, thus our method can incorporate additional types of drug information sources.

To summarize, our study differs from prior related studies in the following aspects: (1) we investigate the use of clinical SEs as key features to predict DDIs. To our knowledge ours is the first study to do so. While Gottlieb *et al.*^15^ used SEs as one of the sources to build predictive models, they did not consider any off-label SEs extracted from FDA Adverse Event Reporting System. (2) we use a label propagation approach for DDI prediction by considering high-order similarity, and propose an integrative label propagation framework by considering drug information from multiple sources for better solutions. The new method not only provides DDI prediction but also ranks and prioritizes multiple drug information sources.

## Materials and Methods

### Preparation of datasets

#### FAERS DDI database

FDA Adverse Event Reporting System (FAERS) is a database that contains information on adverse events submitted to FDA, which is designed to support FDA’s post-marketing safety surveillance program for drugs and therapeutic biological products. Mined from FAERS, TWOSIDES^20^ is a dataset containing only SEs caused by the combination of drugs rather than by any single drugs. In this study, we used the unsafe co-prescriptions from TWOSIDES as known set of DDIs. There are 645 drugs and 63,473 distinct pairwise DDIs in the dataset.

#### Side effect datasets

SIDER is a side effect database of drugs containing information on market medicines and their recorded adverse drug reactions^21^. The information is extracted from package inserts (i.e., drug labels). In this study, we downloaded the entire database from http://sideeffects.embl.de/. There are 996 drugs and 4,192 side effects in the dataset. We called side effects extracted from SIDER as “Label Side Effect”.

SIDER is an important source of known side effects, but the knowledge is limited: (1) Clinical trials are conducted on relatively small patient populations, only common effects can be detected with sufficient confidence to be listed on a drug’s package insert. (2) Effect observed during the clinical trials may be incidental and not actually caused by the drug. OFFSIDES^20^ is a side effect dataset built by mining FAERS system while controlling confounding factors such as concomitant medications, patient demographics, and patient medical histories. There are 1,332 drugs and 10,093 side effects in the dataset. We called side effects extracted from OFFSIDES as “Off-Label Side Effect”.

Merging the drugs from SIDER, OFFSIDES, and TWOSIDES, we obtained 569 drugs with label side effect, off-label side effect, and DDI information. Among 569 drugs, there are 52,416 distinct pairwise DDIs.

#### Chemical structure dataset

Also we extracted chemical structures of the 569 drugs from PubChem^22^, thus we can compare chemical structures information with side effect information.

### Similarity measures

For side effect information, each drug was represented by a binary side effect profile (4,192-dimensional for label side effect and 10,093-dimensional for off-label side effect) whose elements encode for the presence or absence of each of the side effect key words by 1 or 0 respectively.

For chemical structure information, we used a chemical structure fingerprint corresponding to the 881 chemical substructures^23^ defined in the PubChem. Each drug was represented by an 881-dimensional binary profile whose elements encode for the presence or absence of each PubChem substructure by 1 or 0, respectively.

We used Tanimoto coefficient (TC), also known as the Jaccard index, to compute similarities between all the fingerprints. The TC between two fingerprints A and B is defined as the ratio between the number of features in the intersection to the union of both fingerprints: TC(A,B) = |A ⋂ B|/|A ⋃ B|. Then we created matrices so that the rows and columns represent drugs and each cell represents the TC between fingerprint pair of drugs. Drug information from chemical structure, prescription package inserts, and FAERS was thus transferred into chemical similarity, label side effect similarity, and off-label side effect similarity matrices.

### Label propagation algorithm

Label propagation algorithms address the following problem: given an undirected weighted network with *n* nodes where a small portion of them are labeled (e.g., as positive), estimate the labels of the rest unlabeled nodes. In our case, we treated different drugs as nodes on the network, and computed the edge weights on the network with drug similarities evaluated using the method in last section. For each drug, we labelled all other drugs in the network as positive if they are known to have DDI with this drug, and utilized label propagation on such drug network to estimate the possibility that the unlabeled drugs will have DDI with this drug. We didn’t consider negative examples, as in the DDI prediction problem negatives (i.e., the known safe co-prescriptions) are rarely available.

More concretely, we represented the input drug network using an *n* × *n* affinity matrix ***A***, where ***A***_ij_ ≥ 0 is the similarity between drug *i* and *j*. For each drug, we constructed a label vector *y* ∈ {0,1}^n^ over the network, where *y*_i_ = 1 if drug *i* is known to have DDI with the reference drug, and *y*_*i*_ = 0 otherwise. With those notations, label propagation assigns scores (which indicate the possibility that each drug will have DDI with the reference drug) to drug nodes by an iterative procedure which propagates evidence out from positive nodes through the edges in the network. To ensure convergence of the updates, the original affinity matrix ***A*** needs to be normalized so that the row sum is one. In this study, we used Bregmanian Bi-Stochastication (BBS) algorithm^24^ for such normalization and denote the normalized matrix as ***W***. One nice thing of BBS is that the resultant normalized matrix is still symmetric.

Using ***W***, we propagated labels from the labeled drug nodes to the unlabeled nodes. In each propagation iteration, the estimated score of each drug node “absorbs” a portion (*μ*) of the label information from its neighborhood, and retains a portion (1 − *μ*) of its initial label information. The updating rule for node *i* is given by 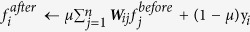. In this formula, 0 < *μ* < 1 is a parameter that determine the influence of a node’s neighbors relative to its provided label. By concatenating the predicted scores for all drug nodes, we can obtain the matrix form of the updating rule^25^ as 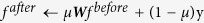. It can be shown that after *t* iterations, the predicted score vector *f  *^*t*^ can be written as 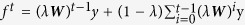. Since ***W***_ij_ ≥ 0, and 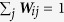, the spectral radius of ***W***, or ρ(***W***) ≤ 1. In additional, 0 < *μ* < 1, thus 
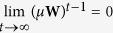
 and 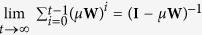 where ***I*** is the identity matrix of order *n*. Therefore, *f*^*t*^ will eventually converge to 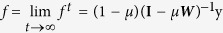.

In our scenario, there are *n* tasks, i.e., we want to predict the DDI profile for each drug. To achieve this, we can first concatenate the initial label vector *y* for each drug into an initial label matrix ***Y***, where its (*i*, *j*)-th entry is 1 if drug *i* interacts with drug *j*, and 0 means there *i*s no known interaction between drug *i* and drug *j*. Then we can get all the DDI predictions in one-shot as ***F*** = (1 − *μ*)(**I** − *μ**W***)^−1^***Y***^26^.

Actually the converged solution for label propagation can also be obtained by minimizing the following objective:


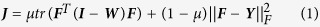


In this formula, *tr*(•) denotes the trace of a matrix, ‖•‖_F_ denotes Frobenius norm of a matrix. The first term of (1) is a smoothness term, which assumes that the prediction should not vary too much on the intrinsic network. In our case, it means that the predicted DDI score for any reference drugs should change smoothly over the drug network. This coincides with our drug similarity assumption: similar drugs tend to have similar DDI effects. The second term of (1) is the fitting term, which restricts the predicted DDI scores to be close to their initial values. The trade-off between those two competing terms is captured by a nonnegative parameter *μ* between 0 and 1. As formula (1) is convex with respect to ***F***, we can get its global optimum by setting the first order derivative of ***J*** with respect to ***F*** to zero. As 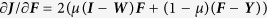. By setting 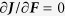, we can still get ***F*** = (1 − *μ*)(**I **− *μ**W***)^−1^***Y***.

### The difference between label propagation and nearest neighbor strategies

In last section, we introduced a label propagation approach for predicting DDIs. Here we compare it to the nearest neighbor strategy. The motivating hypothesis for similarity based DDI prediction is: if drug *i* and drug *j* are similar according to some criteria and drug *k* interacts with drug *i*, then drug *k* will be very likely to interact with drug *j* as well. We first constructed a drug similarity network according to various drugs’ characteristics (chemical structures, label side effects, or off-label side effects), and then spread DDI labels of the drugs on such network.

**Figure 1:**
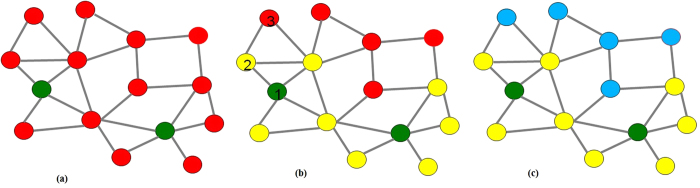
Comparison of nearest neighbor and label propagation strategies. (**a**) toy training data with two known drugs (in green) interacting with the input; (**b**) nearest neighbor method only searches drugs similar to the training drugs (in yellow); (**c**) label propagation method searches the whole drug similarity graph, i.e., search yellow drugs’ nearest neighbors as well (in blue).

To better depict this idea, we constructed a synthetic example in Fig. 1. Figure 1(a) shows a drug network structure, where any two drugs whose similarity is larger than a specific threshold are connected. We colored the nodes according to whether the drugs in the network are known to interact with the reference drug. In this case, the two green drugs are known to interact with the query drug, and the red drugs are the unknown ones that we want to predict. The nearest neighbor algorithms will act as in Fig. 1(b): they search the whole drug space, find the most similar drugs to the green ones (these drugs are plotted in yellow), and predict the yellow ones will interact with the input drug. For example, drug 1 is similar to drug 2, but drug 1 is not similar enough to drug 3. Therefore, only drug 2 is predicted to interact with the input drug. However, if drug 3 is very similar to drug 2 (and also another yellow drug in the figure), it may also have high likelihood of interacting with the input drug. Our proposed approach goes further, acting as Fig. 1(c): it will continue to search yellow drugs’ nearest neighbors as well (in blue), and iterate this process until convergence.

### Label propagation with multiple similarities

In previous sections we discussed the case when we have only one drug similarity matrix. In the real-world applications, we can have multiple drug similarities derived from different data sources (e.g., chemical, label side effect, and off-label side effect). In this case if we only use one drug similarity matrix then the resultant predictions could be biased. It may also suffer from the noise in the focused drug information source. Because of the complex mechanism of the DDIs, their predictions clearly need methods to integrate drug information from multiple sources for better solutions.

Let ***Y*** be the initial DDI matrix and suppose we have *K* symmetrically normalized drug similarity matrices ***W***_1_, ***W***_2_, …, ***W***_K_, where each of them captures the drug similarity from one specific perspective. We would like to obtain DDI predictions ***F*** by solving the following composite optimization problem:


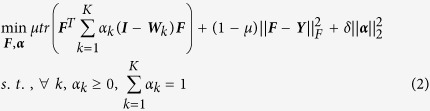


The objective function (2) is derived from objective function (1) by linear combination of individual drug similarity matrices from different data sources. In this formula, *tr*(•) denotes the trace of a matrix, ‖•‖_F_ denotes Frobenius norm of a matrix, and ‖•‖_2_ denotes *l*_2_ norm of a vector. Since we have *K* drug similarity matrices ***W***_*1*_, ***W***_*2*_, …, ***W***_*K*_, we defined and constrained a weight coefficients 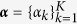 in a simplex, whose *k*-th element is the importance of drug similarity matrix ***W***_*k*_, and the sum of all element of ***α*** is 1. Similar to the definitions in formula (1), the first term of the objective in problem (2) is the prediction smoothness, which means that a good classification function should not change too much between nearby points. The second term of (2) is the fitness term, which means a good classification function should be consistent with the initial label assignment. The trade-off between those two terms is captured by a positive parameter *μ* which between 0 and 1. In the third term of (2), 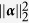 is a regularizer to avoid trivial solutions, and *δ* > 0 is a regularization parameter.

There are two groups of variables, ***F*** and ***α***, in problem (2). Although the objective of problem (2) is not jointly convex with respect to ***F*** and ***α***, it is convex with respect to one group of variables with the other group fixed. Thus, we adopted *Block Coordinate Descent* (*BCD*) schema, which starts by initializing both groups’ variables, and then alternatively solves the optimization problem with respect to one group of variable with the other group fixed. The resultant two sub-problems are:

#### Fix *α,* solve *F*

In this case, the weight vector ***α*** is given: the *k*-th element of ***α*** corresponds to the importance of the *k*-th similarity matrix, and the sum of all elements in ***α*** is 1. This is the same problem as the single similarity matrix case if we make 
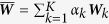
. Thus the solution of this iterative step is 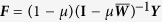. In our experiment, we don’t have any prior knowledge on different sources. Therefore, we initialize ***α*** from a uniform distribution (i.e., assuming all similarity matrices are equally weighted in the beginning).

#### Fix *F*, solve ***α***

When F is fixed, the problem in formula (2) becomes


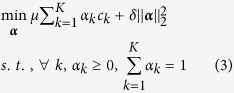


where *c*_*k*_ = *tr*(***F***^*T*^(***I*** − ***W***_*k*_)***F***), which is a standard *Quadratic Programming (QP)* problem.

Here, we reformulated this problem to facilitate more efficient solutions. For notational convenience, we denoted ***C*** = (*c*_*1*_, *c*_*2*_, …, *c*_*k*_)^T^. we first rewrote the objective of problem (3) as


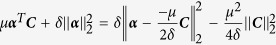


As 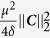 is a constant with respect to ***α*** and *δ* > 0, (3) can be rewritten as


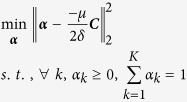


where *μ* is the influence parameter and *δ* is the regularization parameter as in (2) and (3). This is a Euclidean projection problem under the simplex constraint and can be solved by the algorithm in^27^,^28^ (as in our experiments).

After the alternating optimization procedure converges, we obtained both the predicted DDI matrix ***F*** and the weight coefficient vector ***α*** that can be used to rank and prioritize multiple similarity matrices.

## Results

### Performance evaluation

A hold-out validation was carried out by dividing the initial DDI dataset into training and testing subsets. To ensure the validity of the test cases, we held out all the DDIs associated with a fixed percentage of the drugs, rather than holding out DDIs directly. This validation setting mimics a real-world situation of the drug discovery: once drugs without any interaction information come, a computational method should provide the DDI prediction based on properties of both these drugs and training drugs which exist in the current system. To be specific, we randomly selected a fixed percentage (i.e., 15%, 25%, 50%, 75%, and 85%) of drugs for testing, and moved all DDIs associated with these drugs to the testing set. Then we constructed the models with the remaining DDIs as the training set. The model parameters were tuned with cross validation based on the training set. Models were tested on the testing set only after all model parameter tuning has been done. For each testing, we repeated the hold-out validation experiment 50 times with different random divisions of the DDI dataset, and computed the mean and the standard deviation of the *Area Under the Receiver Operating Characteristic Curve* (*AUROC*) as well as the *Area Under the Precision-Recall Curve* (*AUPR*) over the 50 repetitions. In the ROC and PR analytics, we utilized DDI interactions from TWOSIDES as reference positives, and the complement set of TWOSIDES DDI interactions as reference negatives.

In the experiment, we compared seven DDI prediction methods: (1) **Nearest Neighbor with Chemical Similarity** (NN-Chemical) that identifies novel DDIs by using the nearest neighbor similarity to drugs involved in established DDIs. The similarity metric used in this method is based on chemical structures. A similar method was published in reference^10^. (2) **Nearest Neighbor with Label Side Effect Similarity** (NN-LabelSE) that identifies novel DDIs by using the nearest neighbor similarity to drugs involved in established DDIs. The similarity metric used in this method is based on drug label side effect information. (3) **Nearest Neighbor with Off-Label Side Effect Similarity** (NN-OffLabelSE) that identifies novel DDIs by using the nearest neighbor similarity to drugs involved in established DDIs. The similarity metric used in this method is based on FAERS off-label side effect information. (4) **Label Propagation with Chemical Similarity** (LP-Chemical) that predicts novel DDIs by propagating established DDI information iteratively through the network (details described in “Label propagation algorithm” section). Weight between two nodes (i.e., drugs) in the network is measured by chemical structure similarity. (5) **Label Propagation with Label Side Effect Similarity** (LP-LabelSE) that predicts novel DDIs by propagating established DDI information iteratively through the network. Weight between two nodes in the network is measured by drug label side effect similarity. (6) **Label Propagation with Off-Label Side Effect Similarity** (LP-OffLabelSE) that predicts novel DDIs by propagating established DDI information iteratively through the network. Weight between two nodes in the network is measured by FAERS off-label side effect similarity. (7) **Label Propagation by Integrating All Similarities** (LP-AllSim) that predicts novel DDIs by integrating label propagation processes of multiple drug similarity networks (details described in “Label propagation with multiple similarities” section). In this study, we integrated networks derived from chemical structure, label side effect, and off-label side effect information sources. The comparisons of the hold-out validation assessed by AUROC are summarized in Table 1 and by AUPR are summarized in Table 2.

Several observations can be made from Table 1: (1) Our proposed label propagation algorithm boosted the DDI prediction performance. With the similarity from the same information source, label propagation based methods obtained much higher AUROC scores than nearest neighbor based methods (e.g., at testing percentage of 15%, NN-Chemical achieved averaged AUROC of 0.6951 and LP-Chemical achieved average AUROC of 0.8676). (2) Side effect information is an effective source for constructing drug network when applying the label propagation algorithm, as LP-LabelSE and LP-OffLabelSE obtained higher AUROC than LP-Chemical. For example, with 15% data testing, LP-LabelSE and LP-OffLabelSE achieved averaged AUROC of 0.8907 and 0.9219 respectively, and LP-Chemical achieved average AUROC of 0.8676. (3) Our proposed LP-AllSim method effectively integrated multiple similarities. For DDI prediction, if we only use one drug information then the resultant predictions could be biased. It may also suffer from the noise in the focused drug information source. At all percentages for testing data, LP-AllSim outperformed both the label propagation methods that use a single similarity source (i.e., LP-Chemical, LP-LabelSE, and LP-OffLabelSE) and the methods that use nearest neighbor algorithmic framework (i.e., NN-Chemical, NN-LabelSE, and NN-OffLabelSE). (4) Across different testing percentages, the higher the percentage (i.e., the less known DDIs as training) the lower the AUROC scores.

In Table 1, the AUROC scores only decrease less than 5% for all DDI prediction methods, even the testing percentage jumps from 15% to 85%. It does not mean DDI prediction is an easy task and a small DDI training data is enough to build a reliable model. For ROC analysis, a large change in the number of false positives may only lead to a small change in the false positive rate. Therefore, AUROC scores can present an over optimistic view of an algorithm’s performance for the highly skewed data^29^. In this study, we also provided a precision-recall analysis as a supplement to the ROC analysis.

As shown in Table 2, AUPR scores of all methods decrease at least 10% for all DDI prediction methods, when the testing rate increases from 15% to 85%. AUPR provides a quantitative assessment of how well, on average, predicted scores of true interactions are separated from predicted scores of true non-interactions. Thus it holds biological significance in practice: among the best ranked predictions that could potentially be experimentally tested, what proportion of true positives is present. Compare to NN-Chemical, NN-LabelSE, and NN-OffLabelSE, the large improvement in AUPRs suggests that the top ranked DDIs found by our methods are more likely to be correct. Even with testing percentage 50%, LP-AllSim still achieves AUPR score that is higher than 0.70. The observation indicates that LP-AllSim is more robust to different performance measures and has higher potential to be useful in real-world pharmaceutical applications.

Table S1 lists the best influence parameter *μ* we used in the experiments for all label propagation algorithms. LP-AllSim also has a regularization parameter *δ*, which is not sensitive to test percentage values. Thus we set *δ* as 1 for all LP-AllSim experiments. Table S1 show that the higher testing percentage value (i.e., the less training data), the larger the best influence parameter *μ*. This observation indicates an important property of the propagation algorithms: when there is little training data, the predictions depend more on the geometric structure of the entire dataset, rather than just the training data. It is also the reason that label propagation strategy outperforms nearest neighbor strategy.

### Data source comparison

Table 1 and Table 2 clearly show that clinical side effect information is more important than chemical structure information. From another perspective, LP-AllSim integrated chemical structure, label side effect, and off-label side effect information sources. Besides DDI prediction, the weight vector ***α*** derived from LP-AllSim is interpretable: the *i*-th element of ***α*** corresponds to the importance of the *i*-th data source, and the sum of all elements of ***α*** is 1. The weights of each data source are summarized in Table 3. Table 3 shows that compared to chemical structure source, side effect sources are more important in DDI prediction. The higher the testing percentage values, LP-AllSim depends more on side effect sources.

To further compare the information sources, we compared the worst-case DDI prediction performance by using chemical structure, label side effect, or off-label side effect information. Our hypothesis is to directly use drug-drug similarity scores as the DDI predictions, and evaluate their performance by using all known DDIs as the testing set (i.e., we didn’t use any known DDI as training set). In the experiment, chemical structure similarity only achieves AUROC of 0.5366, which indicate that drugs that have interactions do not necessarily have similar chemical structures. On the other hand, label side-effect similarity and off-label side-effect similarity achieve AUROCs of 0.6108 and 0.6433 respectively. The observation seems to indicate that drugs having interactions sometime have overlapping serious side effects. The analysis provides a potential guideline to help clinicians to rule out unsafe co-prescriptions.

### Novel predictions and case studies

We applied our LP-AllSim algorithm to predict new DDIs between all 1626 drugs with one or more side effect profiles. Among all the 1626 drugs, 702 have both label side effect information and off-label side effect information, 294 have only label side effect information, and 630 have only the off-label side effect information. We extracted chemical structures of all 1626 drugs from PubChem, thus each drug has at least two data sources. Among the 1,321,125 drug pairs, 63,468 pairs are identified as DDIs from TWOSIDES^20^. We used all the 63,468 pairwise DDIs as the training data, and provided DDI prediction scores for the remaining 1,257,657 drug pairs. For pairs of drugs with all the chemical structure, label side effect, and off-label side effect information, we used LP-AllSim algorithm to integrate all three data sources. Otherwise, we used LP-AllSim algorithm to integrate two data sources (i.e., chemical structure and label side effect, or chemical structure and off-label side effect). We selected a cutoff for the ranked list of predictions according to the best F1-measure obtained from cross validation, thus obtaining 145,068 predicted DDIs from the 1,257,657 drug pairs. All DDIs prediction results are available in http://astro.temple.edu/~tua87106/ddi.html.

We validated our novel predictions by comparing the resulting DDI prediction to known interactions from DrugBank^30^. DrugBank DDIs are extracted from drug’s package inserts (accurate but far from complete), which can be used as independent validation sources. Of all the drug pairs, 1,892 DDIs are found only in DrugBank, but not found in TWOSIDES. The mean and standard deviation of the 1,892 DDI prediction scores are 0.3932 ± 0.1431, which are significantly larger than those of the other 1,255,765 drug pairs (0.0717 ± 0.0996). Besides that, of the 1,892 DrugBank DDIs, 1,876 (99.15%) DDIs are included in our 145,068 predicted DDIs.

As an example, DDI predictions were analyzed between antihypertensive drugs and anti-inflammatory drugs. Table S2 (a) shows the prediction scores between Nonsteroidal anti-inflammatory drugs (NSAIDs) (e.g., ibuprofen, aspirin, and naproxen) and Angiotensin-Converting Enzyme (ACE) Inhibitors (e.g., benazepril, lisinopril, and ramipril)/Angiotensin II Receptor Blockers (ARBs) (e.g., candesartan, eprosartan, and valsartan) antihypertensive drugs. The mean and standard deviation of the 167 predictions are 0.3131 ± 0.0933. And of the 167 predictions, 152 (91.02%) drug pairs are determined as DDIs using the cutoff value described above. Table S2 (b) shows that the prediction scores between NSAIDs and Calcium Channel Blockers (CCBs) (e.g., amlodipine, diltiazem, and felodipine)/Central-Acting Agents (CAAs) (e.g., methyldopa, and clonidine) antihypertensive drugs. The mean and standard deviation of the 88 predictions are 0.1221 ± 0.0647, which are significantly lower than those of predictions between NSAIDs and ACE/ARBs. Table S2 shows that when co-prescribing NSAIDs, hypertensive patients might take CCBs or CAAs antihypertensive drugs instead of ACE or ARBs to avoid potential adverse DDIs. This could be partially explained with the following reasons. NSAIDs inhibit prostaglandin-mediated vasodilation and promote salt and water retention. Both of these mechanisms contributing to NSAIDs partially reverse the effects ACE and ARBs, whose mechanism depends on modulating prostaglandins, renin, or sodium and water balance. In contrast, NSAIDs do not interact with CCBs and CAAs whose actions are apparently unrelated with renal/extrarenal production of prostaglandin^31^. The mechanism of action (MOA) has also been verified by a recent large-scale clinical study^32^.

Another example we analyzed is DDI predictions between cholesterol-lowering statin drugs and antibiotics. Table S3 shows the prediction scores between statin drugs (e.g., atorvastatin, lovastatin, and simvastatin) and clarithromycin/erythromycin. Of the 10 drug pairs included in our predictions, 9 drug pairs are predicted as DDIs. In addition, the only drug pairs which have been predicted as non-DDIs (i.e., atorvastatin and erythromycin lactobionate) gain a relatively high prediction score (i.e., 0.184649), showing that they still have some possibilities to interact with each other. Table S3 indicates that doctors might avoid ordering clarithromycin and erythromycin for older patients who take cholesterol-lowering statin drugs. This could be partially explained with the following reasons. Clarithromycin and erythromycin that inhibit the liver enzyme cytochrome P450 isoenzyme 3A4, and the inhibition might increase statin concentration in the blood, which can cause muscle or kidney damage, and even death. The MOA has been well documented and clinically verified by a recent population-based cohort study^33^.

As a third example, DDI predictions were analyzed between Selective Serotonin Reuptake Inhibitor (SSRI) antidepressants and hydrocodone (a commonly used antitussive). Table S4 shows that the prediction scores between SSRI medications (including citalopram, fluoxetine, fluvoxamine, paroxetine, and sertraline) and hydrocodone. All the 5 drug pairs were predicted as DDIs. Table S4 indicates that hydrocodone may interact with SSRI medications. Our predictions are in agreement with a clinical study^34^ which shows that co-prescription of hydrocodone and SSRI medications may result in serotonin syndrome, a potentially life threatening drug reaction, that causes the body to have too much serotonin, a chemical produced by nerve cells.

## Discussion

In this study, we have proposed an integrative label propagation framework to predict DDIs by integrating label side effects, off-label side effects, and chemical structures. A systematic comparison of the experimental results shows (1) side effect profiles are more predictive features than chemical structures in DDI prediction. It greatly benefits from the fact that clinical side effects are human phenotypic data obviating translation issues. (2) label propagation algorithm boosted the DDI prediction by considering high-order relationships between drugs. (3) our proposed integrative label propagation algorithm effectively integrated multiple drug properties and outperformed competitors. Furthermore, we applied the proposed algorithm to all known drugs which have one or more side effect profiles and obtained 145,068 predicted DDIs. These predicted DDIs can be leveraged for clinical surveillance and real-world drug discovery.

There are some limitations in our study:

(1) Although SE information is more predictive than chemical structures in our DDI prediction experiments, SEs are usually unavailable for clinical candidates. For example, label SEs are detected during the phases of the clinical trials, and off-label SEs are accumulated during the post-market surveillance. For clinical candidates which at the early stages of drug development, the only available information is chemical structures. In future work, we may consider to map compound structure of clinical candidates to possible SEs via quantitative structure-activity relationship (QSAR) models and then predict DDIs based on inferred SEs. A similar idea was introduced by DRoSEf^17^, a methodology of drug repositioning based on the side-effectome.

(2) We used 4,192-dimensional label SE vector and 10,093-dimensional off-label SE vector to quantify each drug. Since some SE terms are similar (e.g., “muscle stiffness” and “muscle tightness”, “ear discomforts” and “ear pain”), multiple synonyms could cause biases in SE profiling. Of the 569 drugs in our evaluation experiments, the chance of two drugs share a group of very similar SE terms is rare; and Tanimoto coefficient (which we used as metric to measure SE similarities) is not sensitive to the dimensionality problem (more details in Supplementary Section 2). Thus, the multiple-name problem doesn’t have a significant impact on the SE similarity measurement. In future work, we will investigate the synonyms among SE terms. We may consider to measure semantic similarity between SE terms, remove redundant SE terms, and build more robust SE profiles to support our DDI predictions.

(3) In this study, we used the unsafe co-prescriptions from TWOSIDES as known set of DDIs. However, TWOSIDES is directly derived from FDA Adverse Event Reporting System (FAERS), which contains some false positives (i.e., drug pairs included in TWOSIDES don’t interact). When we utilized TWOSIDES as known interactions to build predictive models, the false-positive noises may affect the predictions. Fortunately, our proposed label propagation algorithm is robust to these false-positive noises. The objective function (formula (1) in the section “label propagation algorithm”) of our method contains two terms (i.e., smoothness term and fitting term) and these two competing terms is captured by a nonnegative tradeoff parameter *μ* between 0 and 1. The larger the parameter *μ*, the more the objective function depends on the structure of the drug network (which built from either chemical structure or side-effect profiles). Thus as long as the data structure is reliable enough (where we use chemical compound and side-effects), the label propagation process can even correct some of the false-positive label noises.

(4) In the experiment, we only predicted whether two drugs interact or not, without providing the reasons by which the drugs interact. In future work, we may extend our model to link specific reasons to DDI candidates. In this case, the “label” is no longer a simple “interaction”, but interaction with specific reasons. In other words, we have multiple types of labels to propagate, where each type of labels corresponds to interactions with a specific reason. In machine learning, this type of problem is usually referred to as multi-label learning. The proposed label propagation method does have the flexibility of handling multiple labels, where the whole procedure is equivalent to propagation on every specific type of label independently.

## Additional Information

**How to cite this article**: Zhang, P. *et al.* Label Propagation Prediction of Drug-Drug Interactions Based on Clinical Side Effects. *Sci. Rep.* **5**, 12339; doi: 10.1038/srep12339 (2015).

## Supplementary Material

Supplementary Information

## Figures and Tables

**Table 1:** Comparison of DDI prediction methods according to AUROC at different testing percentages.

**Methods**	**15%**	**25%**	**50%**	**75%**	**85%**
NN-Chemical	0.6951+/−0.0031	0.6949+/−0.0021	0.6910+/−0.0016	0.6838+/−0.0017	0.6805+/−0.0017
NN-LabelSE	0.7359+/−0.0018	0.7348+/−0.0019	0.7304+/−0.0016	0.7288+/−0.0016	0.7241+/−0.0020
NN-OffLabelSE	0.7557+/−0.0017	0.7500+/−0.0023	0.7436+/−0.0015	0.7397+/−0.0015	0.7390+/−0.0019
LP-Chemical	0.8676+/−0.0015	0.8654+/−0.0012	0.8603+/−0.0007	0.8515+/−0.0009	0.8415+/−0.0015
LP-LabelSE	0.8907+/−0.0014	0.8880+/−0.0010	0.8816+/−0.0007	0.8713+/−0.0007	0.8642+/−0.0014
LP-OffLabelSE	0.9219+/−0.0012	0.9194+/−0.0010	0.9115+/−0.0009	0.8994+/−0.0009	0.8888+/−0.0012
LP-AllSim	0.9258+/−0.0011	0.9233+/−0.0009	0.9156+/−0.0008	0.9033+/−0.0008	0.8921+/−0.0012

**Table 2:** Comparison of DDI prediction methods according to AUPR at different testing percentages.

**Methods**	**15%**	**25%**	**50%**	**75%**	**85%**
NN-Chemical	0.5182+/−0.0018	0.4932+/−0.0020	0.4067+/−0.0015	0.3810+/−0.0013	0.3667+/−0.0014
NN-LabelSE	0.5537+/−0.0024	0.5137+/−0.0019	0.4549+/−0.0016	0.4038+/−0.0016	0.3791+/−0.0015
NN-OffLabelSE	0.5780+/−0.0022	0.5412+/−0.0026	0.4710+/−0.0015	0.4331+/−0.0016	0.3924+/−0.0014
LP-Chemical	0.6128+/−0.0040	0.6026+/−0.0026	0.5876+/−0.0016	0.5545+/−0.0022	0.4726+/−0.0025
LP-LabelSE	0.6567+/−0.0035	0.6481+/−0.0021	0.6066+/−0.0019	0.5826+/−0.0023	0.5323+/−0.0028
LP-OffLabelSE	0.7195+/−0.0031	0.7189+/−0.0019	0.6827+/−0.0022	0.6477+/−0.0026	0.6349+/−0.0031
LP-AllSim	0.7292+/−0.0032	0.7282+/−0.0021	0.7052+/−0.0022	0.6736+/−0.0025	0.6501+/−0.0034

**Table 3:** LP-AllSim derived weights for chemical structure, label side effect, and off-label side effect information sources in experiments at different testing percentages.

**Sources**	**15%**	**25%**	**50%**	**75%**	**85%**
chemical structure	0.2451+/−0.0035	0.1417+/−0.0021	0.0264+/−0.0012	0.0000+/−0.0003	0.0000+/−0.0000
label side effect	0.3412+/−0.0047	0.3464+/−0.0018	0.3609+/−0.0012	0.3505+/−0.0009	0.2947+/−0.0008
off−label side effect	0.4137+/−0.0041	0.5119+/−0.0019	0.6127+/−0.0016	0.6495+/−0.0006	0.7055+/−0.0010

## References

[b1] Percha B. & Altman R. B. Informatics confronts drug-drug interactions. Trends Pharmacol Sci 34, 178–184 (2013). 23414686 10.1016/j.tips.2013.01.006 PMC3808975

[b2] Juurlink D. N., Mamdani M., Kopp A., Laupacis A. & Redelmeier D. A. Drug-drug interactions among elderly patients hospitalized for drug toxicity. JAMA 289, 1652–1658 (2003). 12672733 10.1001/jama.289.13.1652

[b3] van Leeuwen R. W., Swart E. L., Boom F. A., Schuitenmaker M. S. & Hugtenburg J. G. Potential drug interactions and duplicate prescriptions among ambulatory cancer patients: a prevalence study using an advanced screening method. BMC Cancer 10, 679; 10.1186/1471-2407-10-679 (2010). 21144049 PMC3013087

[b4] Kuhn M. *et al.* STITCH 2: an interaction network database for small molecules and proteins. Nucleic Acids Res 38, D552–D556 (2010). 19897548 10.1093/nar/gkp937 PMC2808890

[b5] He L., Yang Z., Zhao Z., Lin H. & Li Y. Extracting drug-drug interaction from the biomedical literature using a stacked generalization-based approach. PLoS One 8, e65814; 10.1371/journal.pone.0065814 (2013). 23785452 PMC3681788

[b6] Duke J. D. *et al.* Literature based drug interaction prediction with clinical assessment using electronic medical records: novel myopathy associated drug interactions. PLoS Comput Biol 8, e1002614; 10.1371/journal.pcbi.1002614 (2012). 22912565 PMC3415435

[b7] Noren G. N., Sundberg R., Bate A. & Edwards I. R. A statistical methodology for drug-drug interaction surveillance. Stat Med 27, 3057–3070 (2008). 18344185 10.1002/sim.3247

[b8] Tatonetti N. P. *et al.* Detecting drug interactions from adverse-event reports: interaction between paroxetine and pravastatin increases blood glucose levels. Clin Pharmacol Ther 90, 133–142 (2012). 10.1038/clpt.2011.83 PMC3216673 21613990

[b9] Tatonetti N. P., Fernald G. H. & Altman R. B. A novel signal detection algorithm for identifying hidden drug-drug interactions in adverse event reports. J Am Med Inform Assoc 19, 79–85 (2012). 21676938 10.1136/amiajnl-2011-000214 PMC3240755

[b10] Vilar S. *et al.* Drug-drug interaction through molecular structure similarity analysis. J Am Med Inform Assoc 19, 1066–1074 (2012). 22647690 10.1136/amiajnl-2012-000935 PMC3534468

[b11] Luo H. *et al.* DDI-CPI, a server that predicts drug–drug interactions through implementing the chemical–protein interactome. Nucl. Acids Res 42, W46–W52 (2014). 24875476 10.1093/nar/gku433 PMC4086096

[b12] Vilar S., Uriarte E., Santana L., Tatonetti N. P. & Friedman C. Detection of drug-drug interactions by modeling interaction profile fingerprints. PLoS ONE 8, e58321; 10.1371/journal.pone.0058321 (2013). 23520498 PMC3592896

[b13] Cami A., Manzi S., Arnold A. & Reis B. Y. Pharmacointeraction network models predict unknown drug-drug interactions. PLoS ONE 8, e61468; 10.1371/journal.pone.0061468 (2013). 23620757 PMC3631217

[b14] Takarabe M., Shigemizu D., Kotera M., Goto S. & Kanehisa M. Network-based analysis and characterization of adverse drug-drug interactions. J Chem Inf Model 51, 2977–2985 (2011). 21942936 10.1021/ci200367w

[b15] Gottlieb A., Stein G. Y., Oron Y., Ruppin E. & Sharan R. INDI: a computational framework for inferring drug interactions and their associated recommendations. Mol Syst Biol 8, 592; 10.1038/msb.2012.26 (2012). 22806140 PMC3421442

[b16] Campillos M., Kuhn M., Gavin A. C., Jensen L. J. & Bork P. Drug target identification using side-effect similarity. Science 321, 263–266 (2008). 18621671 10.1126/science.1158140

[b17] Yang L. & Agarwal P. Systematic drug repositioning based on clinical side-effects. PLoS ONE 6, e28025; 10.1371/journal.pone.0028025 (2011). 22205936 PMC3244383

[b18] Liu Z. *et al.* Translating clinical findings into knowledge in drug safety evaluation–drug induced liver injury prediction system (DILIps). PLoS Comput Biol 7, e1002310; 10.1371/journal.pcbi.1002310 (2011). 22194678 PMC3240589

[b19] Duran-Frigola M. & Aloy P. Recycling side-effects into clinical markers for drug repositioning. Genome Med 4, 3; 10.1186/gm302 (2012). 22283977 PMC3334551

[b20] Tatonetti N. P., Ye P. P., Daneshjou R. & Altman R. B. Data-driven prediction of drug effects and interactions. Sci Transl Med 4, 125ra31; 10.1126/scitranslmed.3003377 (2012). PMC3382018 22422992

[b21] Kuhn M., Campillos M., Letunic I., Jensen L. J. & Bork P. A side effect resource to capture phenotypic effects of drugs. Mol Syst Biol 6, 343; 10.1038/msb.2009.98 (2010). 20087340 PMC2824526

[b22] Wang Y. *et al.* PubChem: a public information system for analyzing bioactivities of small molecules. Nucleic Acids Res 37, W623–W633 (2009). 19498078 10.1093/nar/gkp456 PMC2703903

[b23] PubChem substructure fingerprint. Available at: ftp://ftp.ncbi.nlm.nih.gov/pubchem/specifications/pubchem_fingerprints.pdf (Accessed: 17th January 2015).

[b24] Wang F., Li P., Konig A. C. & Wan M. Improving clustering by learning a bi-stochastic data similarity matrix. Knowl Inf Syst 32, 351–382 (2010).

[b25] Vanunu O., Magger O., Ruppin E., Shlomi T. & Sharan R. Associating genes and protein complexes with disease via network propagation. PLoS Comput Biol 6, e1000641; 10.1371/journal.pcbi.1000641 (2010). 20090828 PMC2797085

[b26] Wang F. & Zhang C. Label propagation through linear neighborhoods. International Conference on Machine Learning (ICML). 985–992 (2006).

[b27] Duchi J., Shalev-Shwartz S., Singer Y. & Chandra T. Efficient projections onto the l1-ball for learning in high dimensions. International conference on Machine learning (ICML). 272–279 (2008).

[b28] Chen Y. & Ye X. Projection onto a simplex. arXiv :1101.6081 (2011).

[b29] Davis J. & Goadrich M. The relationship between precision-recall and ROC curves. International Conference on Machine Learning (ICML). 233–240 (2006).

[b30] Knox C. *et al.* DrugBank 3.0: a comprehensive resource for ‘omics’ research on drugs. Nucleic Acids Res 39, D1035–D1041 (2011). 21059682 10.1093/nar/gkq1126 PMC3013709

[b31] Polonia J. Interaction of antihypertensive drugs with anti-inflammatory drugs. Cardiology 88, 47–51 (1997). 10.1159/000177507 9397294

[b32] Fournier J. P. *et al.* Non-steroidal anti-inflammatory drugs (NSAIDs) and hypertension treatment intensification: a population-based cohort study. Eur J Clin Pharmacol 68, 1533–1540 (2012). 22527348 10.1007/s00228-012-1283-9

[b33] Patel A. M. *et al.* Statin toxicity from macrolide antibiotic coprescription: a population-based cohort study. Ann Intern Med 158, 869–876 (2013). 23778904 10.7326/0003-4819-158-12-201306180-00004

[b34] Gnanadesigan N., Espinoza R. T. & Smith R. L. The serotonin syndrome. N Engl J Med 352, 2454–2456 (2005). 15948273

