# Supplementary material for: Label Propagation Prediction of Drug-Drug Interactions Based on Clinical Side Effects
Source: Sci Rep. 2015 Jul 21;5:12339. doi: 10.1038/srep12339 (PMC5387872; doi:10.1038/srep12339)
Supplement: Supplementary Information [file srep12339-s1.doc]

**Label Propagation Prediction of Drug-Drug Interactions Based on Clinical Side Effects**

Ping Zhang^1, *^, Fei Wang^2^, Jianying Hu^1^, Robert Sorrentino^1^

1 Healthcare Analytics Research, IBM T. J. Watson Research Center, Yorktown Heights, USA

2 Computer Science and Engineering, University of Connecticut, Storrs, USA

*Corresponding author

Email addresses:

PZ: pzhang@us.ibm.com

**Supplementary Information**

**Section 1: Supplementary Tables (S1 – S4)**

**Table S1 - Influence parameter *µ* of all label propagation algorithms**

| Methods | 15% | 25% | 50% | 75% | 85% |
| --- | --- | --- | --- | --- | --- |
| LP-Chemical | 0.08 | 0.16 | 0.50 | 0.60 | 0.70 |
| LP-LabelSE | 0.16 | 0.30 | 0.60 | 0.90 | 0.95 |
| LP-OffLabelSE | 0.16 | 0.30 | 0.60 | 0.90 | 0.95 |
| LP-AllSim | 0.16 | 0.30 | 0.60 | 0.80 | 0.90 |

**Table S2 - Prediction scores between antihypertensive drugs and anti-inflammatory drugs**

(a) prediction scores between Nonsteroidal anti-inflammatory drugs (NSAIDs) and Angiotensin-Converting Enzyme (ACE) Inhibitors/Angiotensin II Receptor Blockers (ARBs) antihypertensive drugs; (b) prediction scores between Nonsteroidal anti-inflammatory drugs (NSAIDs) and Calcium Channel Blockers (CCBs)/Central-Acting Agents (CAAs) antihypertensive drugs.

(a) In the table, Nonsteroidal anti-inflammatory drugs (NSAIDs) include ibuprofen, aspirin, naproxen, celecoxib, sulindac, oxaprozin, diflunisal, piroxicam, indomethacin, etodolac, meloxicam, naproxen, nabumetone, ketorolac tromethamine, and diclofenac; Angiotensin-Converting Enzyme (ACE) Inhibitors include benazepril, captopril, enalapril, fosinopril, lisinopril, perindopril erbumine, perindopril, quinapril, ramipril, and trandolapril; Angiotensin II Receptor Blockers (ARBs) include candesartan cilexetil, candesartan, eprosartan, irbesartan, losartan, olmesartan, telmisartan, and valsartan.

| DRUG1_ID | DRUG1_NAME | DRUG2_ID | DRUG2_NAME | PRED_SCORE | PRED_LABEL |
| --- | --- | --- | --- | --- | --- |
| CID000003672 | ibuprofen | CID000005525 | trandolapril | 0.538574 | Y |
| CID000003937 | lisinopril | CID000084003 | ketorolac tromethamine | 0.519735 | Y |
| CID000002662 | celecoxib | CID000005525 | trandolapril | 0.517754 | Y |
| CID000002311 | benazepril | CID000003032 | diclofenac | 0.480012 | Y |
| CID000003059 | diflunisal | CID000158781 | olmesartan | 0.476709 | Y |
| CID000005650 | valsartan | CID000084003 | ketorolac tromethamine | 0.4677 | Y |
| CID000003059 | diflunisal | CID000060878 | eprosartan | 0.466166 | Y |
| CID000003059 | diflunisal | CID000060183 | perindopril erbumine | 0.464665 | Y |
| CID000003032 | diclofenac | CID000005525 | trandolapril | 0.461395 | Y |
| CID000004051 | meloxicam | CID000005525 | trandolapril | 0.460148 | Y |
| CID000005038 | ramipril | CID000084003 | ketorolac tromethamine | 0.459072 | Y |
| CID000002540 | candesartan cilexetil | CID000003059 | diflunisal | 0.45828 | Y |
| CID000003749 | irbesartan | CID000004614 | oxaprozin | 0.457879 | Y |
| CID000003961 | losartan | CID000084003 | ketorolac tromethamine | 0.457399 | Y |
| CID000003059 | diflunisal | CID000060879 | eprosartan | 0.456171 | Y |
| CID000001301 | naproxen | CID000005525 | trandolapril | 0.451011 | Y |
| CID000003222 | enalapril | CID000084003 | ketorolac tromethamine | 0.449292 | Y |
| CID000003059 | diflunisal | CID000065999 | telmisartan | 0.4445 | Y |
| CID000003059 | diflunisal | CID000005038 | ramipril | 0.432548 | Y |
| CID000004052 | meloxicam | CID000005525 | trandolapril | 0.431533 | Y |
| CID000005525 | trandolapril | CID000084003 | ketorolac tromethamine | 0.430931 | Y |
| CID000003059 | diflunisal | CID000005650 | valsartan | 0.430352 | Y |
| CID000002541 | candesartan | CID000004614 | oxaprozin | 0.427767 | Y |
| CID000005352 | sulindac | CID000158781 | olmesartan | 0.426338 | Y |
| CID000003059 | diflunisal | CID000003961 | losartan | 0.426137 | Y |
| CID000004614 | oxaprozin | CID000158781 | olmesartan | 0.422292 | Y |
| CID000003059 | diflunisal | CID000005525 | trandolapril | 0.42118 | Y |
| CID000002541 | candesartan | CID000004856 | piroxicam | 0.418748 | Y |
| CID000005352 | sulindac | CID000060878 | eprosartan | 0.417369 | Y |
| CID000003059 | diflunisal | CID000003222 | enalapril | 0.413833 | Y |
| CID000001301 | naproxen | CID000003937 | lisinopril | 0.413549 | Y |
| CID000002311 | benazepril | CID000004051 | meloxicam | 0.413539 | Y |
| CID000002244 | aspirin | CID000060879 | eprosartan | 0.412641 | Y |
| CID000002550 | captopril | CID000003308 | etodolac | 0.407965 | Y |
| CID000004614 | oxaprozin | CID000060878 | eprosartan | 0.407051 | Y |
| CID000002550 | captopril | CID000004614 | oxaprozin | 0.406255 | Y |
| CID000003419 | fosinopril | CID000004051 | meloxicam | 0.401505 | Y |
| CID000003672 | ibuprofen | CID000060879 | eprosartan | 0.39716 | Y |
| CID000004051 | meloxicam | CID000060184 | perindopril | 0.395958 | Y |
| CID000004856 | piroxicam | CID000158781 | olmesartan | 0.39078 | Y |
| CID000002541 | candesartan | CID000005352 | sulindac | 0.386196 | Y |
| CID000003749 | irbesartan | CID000084003 | ketorolac tromethamine | 0.379721 | Y |
| CID000005352 | sulindac | CID000060183 | perindopril erbumine | 0.378039 | Y |
| CID000002662 | celecoxib | CID000060879 | eprosartan | 0.377452 | Y |
| CID000004614 | oxaprozin | CID000060183 | perindopril erbumine | 0.375121 | Y |
| CID000002311 | benazepril | CID000003715 | indomethacin | 0.374194 | Y |
| CID000002244 | aspirin | CID000002540 | candesartan cilexetil | 0.370368 | Y |
| CID000003419 | fosinopril | CID000004409 | nabumetone | 0.368514 | Y |
| CID000002540 | candesartan cilexetil | CID000004614 | oxaprozin | 0.365432 | Y |
| CID000002540 | candesartan cilexetil | CID000005352 | sulindac | 0.365347 | Y |
| CID000004856 | piroxicam | CID000060878 | eprosartan | 0.364553 | Y |
| CID000002540 | candesartan cilexetil | CID000003672 | ibuprofen | 0.363743 | Y |
| CID000001301 | naproxen | CID000005650 | valsartan | 0.358326 | Y |
| CID000001302 | naproxen | CID000060879 | eprosartan | 0.357067 | Y |
| CID000005005 | quinapril | CID000005352 | sulindac | 0.356933 | Y |
| CID000002541 | candesartan | CID000084003 | ketorolac tromethamine | 0.353099 | Y |
| CID000003715 | indomethacin | CID000005525 | trandolapril | 0.35085 | Y |
| CID000002311 | benazepril | CID000003308 | etodolac | 0.350797 | Y |
| CID000003308 | etodolac | CID000158781 | olmesartan | 0.35055 | Y |
| CID000001301 | naproxen | CID000005038 | ramipril | 0.349629 | Y |
| CID000004409 | nabumetone | CID000158781 | olmesartan | 0.348861 | Y |
| CID000002244 | aspirin | CID000060183 | perindopril erbumine | 0.347736 | Y |
| CID000005352 | sulindac | CID000060879 | eprosartan | 0.34594 | Y |
| CID000005005 | quinapril | CID000084003 | ketorolac tromethamine | 0.34529 | Y |
| CID000001301 | naproxen | CID000003961 | losartan | 0.344802 | Y |
| CID000002550 | captopril | CID000084003 | ketorolac tromethamine | 0.344621 | Y |
| CID000003672 | ibuprofen | CID000060183 | perindopril erbumine | 0.344023 | Y |
| CID000003059 | diflunisal | CID000003749 | irbesartan | 0.343833 | Y |
| CID000004614 | oxaprozin | CID000060879 | eprosartan | 0.342947 | Y |
| CID000003308 | etodolac | CID000060878 | eprosartan | 0.341454 | Y |
| CID000001301 | naproxen | CID000003222 | enalapril | 0.341081 | Y |
| CID000005352 | sulindac | CID000065999 | telmisartan | 0.339965 | Y |
| CID000004409 | nabumetone | CID000060878 | eprosartan | 0.339868 | Y |
| CID000002540 | candesartan cilexetil | CID000002662 | celecoxib | 0.338697 | Y |
| CID000003032 | diclofenac | CID000060879 | eprosartan | 0.337353 | Y |
| CID000001301 | naproxen | CID000002311 | benazepril | 0.335997 | Y |
| CID000002541 | candesartan | CID000003059 | diflunisal | 0.335842 | Y |
| CID000004409 | nabumetone | CID000005525 | trandolapril | 0.33014 | Y |
| CID000003715 | indomethacin | CID000158781 | olmesartan | 0.322184 | Y |
| CID000001301 | naproxen | CID000003419 | fosinopril | 0.320952 | Y |
| CID000003308 | etodolac | CID000005525 | trandolapril | 0.320279 | Y |
| CID000002662 | celecoxib | CID000060183 | perindopril erbumine | 0.319096 | Y |
| CID000001302 | naproxen | CID000002540 | candesartan cilexetil | 0.318458 | Y |
| CID000004051 | meloxicam | CID000065999 | telmisartan | 0.311027 | Y |
| CID000002540 | candesartan cilexetil | CID000003032 | diclofenac | 0.30837 | Y |
| CID000002550 | captopril | CID000003059 | diflunisal | 0.307721 | Y |
| CID000065999 | telmisartan | CID000084003 | ketorolac tromethamine | 0.306564 | Y |
| CID000002550 | captopril | CID000004051 | meloxicam | 0.303924 | Y |
| CID000001302 | naproxen | CID000060183 | perindopril erbumine | 0.301123 | Y |
| CID000003419 | fosinopril | CID000004856 | piroxicam | 0.298608 | Y |
| CID000004052 | meloxicam | CID000060879 | eprosartan | 0.2976 | Y |
| CID000003715 | indomethacin | CID000060878 | eprosartan | 0.295089 | Y |
| CID000004856 | piroxicam | CID000060183 | perindopril erbumine | 0.294627 | Y |
| CID000002311 | benazepril | CID000004856 | piroxicam | 0.293437 | Y |
| CID000003032 | diclofenac | CID000060183 | perindopril erbumine | 0.287766 | Y |
| CID000001301 | naproxen | CID000002541 | candesartan | 0.286972 | Y |
| CID000004051 | meloxicam | CID000005005 | quinapril | 0.285256 | Y |
| CID000001301 | naproxen | CID000003749 | irbesartan | 0.280224 | Y |
| CID000004409 | nabumetone | CID000060184 | perindopril | 0.276157 | Y |
| CID000004856 | piroxicam | CID000005525 | trandolapril | 0.269337 | Y |
| CID000002540 | candesartan cilexetil | CID000004052 | meloxicam | 0.269072 | Y |
| CID000002540 | candesartan cilexetil | CID000004856 | piroxicam | 0.267023 | Y |
| CID000003308 | etodolac | CID000060184 | perindopril | 0.264589 | Y |
| CID000003419 | fosinopril | CID000004614 | oxaprozin | 0.259008 | Y |
| CID000003672 | ibuprofen | CID000060878 | eprosartan | 0.25888 | Y |
| CID000003937 | lisinopril | CID000004051 | meloxicam | 0.258492 | Y |
| CID000003749 | irbesartan | CID000004051 | meloxicam | 0.258114 | Y |
| CID000004856 | piroxicam | CID000060879 | eprosartan | 0.252955 | Y |
| CID000002244 | aspirin | CID000060878 | eprosartan | 0.252607 | Y |
| CID000004052 | meloxicam | CID000060183 | perindopril erbumine | 0.252213 | Y |
| CID000004614 | oxaprozin | CID000060184 | perindopril | 0.251403 | Y |
| CID000001301 | naproxen | CID000002550 | captopril | 0.250634 | Y |
| CID000003672 | ibuprofen | CID000158781 | olmesartan | 0.249885 | Y |
| CID000002244 | aspirin | CID000158781 | olmesartan | 0.249772 | Y |
| CID000002311 | benazepril | CID000003059 | diflunisal | 0.248741 | Y |
| CID000001301 | naproxen | CID000005005 | quinapril | 0.246229 | Y |
| CID000002541 | candesartan | CID000004051 | meloxicam | 0.245826 | Y |
| CID000001301 | naproxen | CID000065999 | telmisartan | 0.245659 | Y |
| CID000004856 | piroxicam | CID000060184 | perindopril | 0.243731 | Y |
| CID000001301 | naproxen | CID000060184 | perindopril | 0.238789 | Y |
| CID000002311 | benazepril | CID000004614 | oxaprozin | 0.237636 | Y |
| CID000005352 | sulindac | CID000060184 | perindopril | 0.237179 | Y |
| CID000004052 | meloxicam | CID000158781 | olmesartan | 0.235807 | Y |
| CID000003715 | indomethacin | CID000060879 | eprosartan | 0.23567 | Y |
| CID000002662 | celecoxib | CID000060878 | eprosartan | 0.234726 | Y |
| CID000003308 | etodolac | CID000060183 | perindopril erbumine | 0.232577 | Y |
| CID000002311 | benazepril | CID000084003 | ketorolac tromethamine | 0.232142 | Y |
| CID000002662 | celecoxib | CID000158781 | olmesartan | 0.22799 | Y |
| CID000001302 | naproxen | CID000060878 | eprosartan | 0.224373 | Y |
| CID000002311 | benazepril | CID000005352 | sulindac | 0.223931 | Y |
| CID000004051 | meloxicam | CID000005650 | valsartan | 0.223767 | Y |
| CID000004409 | nabumetone | CID000060183 | perindopril erbumine | 0.222871 | Y |
| CID000003419 | fosinopril | CID000005352 | sulindac | 0.221399 | Y |
| CID000004052 | meloxicam | CID000060878 | eprosartan | 0.219521 | Y |
| CID000002540 | candesartan cilexetil | CID000003715 | indomethacin | 0.219323 | Y |
| CID000003961 | losartan | CID000004051 | meloxicam | 0.212737 | Y |
| CID000004409 | nabumetone | CID000060879 | eprosartan | 0.212374 | Y |
| CID000001302 | naproxen | CID000158781 | olmesartan | 0.212 | Y |
| CID000004051 | meloxicam | CID000005038 | ramipril | 0.211223 | Y |
| CID000003059 | diflunisal | CID000003419 | fosinopril | 0.210873 | Y |
| CID000003308 | etodolac | CID000060879 | eprosartan | 0.209398 | Y |
| CID000003032 | diclofenac | CID000060878 | eprosartan | 0.209214 | Y |
| CID000003032 | diclofenac | CID000158781 | olmesartan | 0.207099 | Y |
| CID000060184 | perindopril | CID000084003 | ketorolac tromethamine | 0.206657 | Y |
| CID000002540 | candesartan cilexetil | CID000003308 | etodolac | 0.206254 | Y |
| CID000003222 | enalapril | CID000004051 | meloxicam | 0.204022 | Y |
| CID000004614 | oxaprozin | CID000005525 | trandolapril | 0.203966 | Y |
| CID000002540 | candesartan cilexetil | CID000004409 | nabumetone | 0.202785 | Y |
| CID000005352 | sulindac | CID000005525 | trandolapril | 0.202331 | Y |
| CID000003715 | indomethacin | CID000060183 | perindopril erbumine | 0.20153 | Y |
| CID000003059 | diflunisal | CID000060184 | perindopril | 0.201216 | Y |
| CID000003419 | fosinopril | CID000084003 | ketorolac tromethamine | 0.200666 | Y |
| CID000001301 | naproxen | CID000002540 | candesartan cilexetil | 0.194807 | N |
| CID000001301 | naproxen | CID000060183 | perindopril erbumine | 0.189479 | N |
| CID000001301 | naproxen | CID000060878 | eprosartan | 0.187898 | N |
| CID000001301 | naproxen | CID000060879 | eprosartan | 0.185416 | N |
| CID000001301 | naproxen | CID000158781 | olmesartan | 0.179447 | N |
| CID000002540 | candesartan cilexetil | CID000004051 | meloxicam | 0.17701 | N |
| CID000002540 | candesartan cilexetil | CID000084003 | ketorolac tromethamine | 0.176916 | N |
| CID000004051 | meloxicam | CID000060183 | perindopril erbumine | 0.172301 | N |
| CID000004051 | meloxicam | CID000060878 | eprosartan | 0.172136 | N |
| CID000004051 | meloxicam | CID000060879 | eprosartan | 0.171733 | N |
| CID000004051 | meloxicam | CID000158781 | olmesartan | 0.167417 | N |
| CID000060183 | perindopril erbumine | CID000084003 | ketorolac tromethamine | 0.166648 | N |
| CID000060878 | eprosartan | CID000084003 | ketorolac tromethamine | 0.166225 | N |
| CID000060879 | eprosartan | CID000084003 | ketorolac tromethamine | 0.151338 | N |
| CID000084003 | ketorolac tromethamine | CID000158781 | olmesartan | 0.148212 | N |

(b) In the table, Nonsteroidal anti-inflammatory drugs (NSAIDs) include ibuprofen, aspirin, naproxen, celecoxib, sulindac, oxaprozin, diflunisal, piroxicam, indomethacin, etodolac, meloxicam, naproxen, nabumetone, ketorolac tromethamine, and diclofenac; Calcium Channel Blockers (CCBs) include amlodipine, diltiazem, felodipine, isradipine, nicardipine, nifedipine, nisoldipine, and verapamil; Central-Acting Agents (CAAs) include clonidine, guanfacine, and methyldopa.

| DRUG1_ID | DRUG1_NAME | DRUG2_ID | DRUG2_NAME | PRED_SCORE | PRED_LABEL |
| --- | --- | --- | --- | --- | --- |
| CID000004052 | meloxicam | CID000004473 | nicardipine | 0.285256 | Y |
| CID000003519 | guanfacine | CID000003715 | indomethacin | 0.267023 | Y |
| CID000003519 | guanfacine | CID000004409 | nabumetone | 0.258114 | Y |
| CID000003715 | indomethacin | CID000003784 | isradipine | 0.252955 | Y |
| CID000001301 | naproxen | CID000003333 | felodipine | 0.248741 | Y |
| CID000003308 | etodolac | CID000003519 | guanfacine | 0.245826 | Y |
| CID000003784 | isradipine | CID000004409 | nabumetone | 0.238789 | Y |
| CID000003715 | indomethacin | CID000004138 | methyldopa | 0.235807 | Y |
| CID000003308 | etodolac | CID000003784 | isradipine | 0.232577 | Y |
| CID000004409 | nabumetone | CID000004473 | nicardipine | 0.194754 | N |
| CID000002520 | verapamil | CID000004051 | meloxicam | 0.191427 | N |
| CID000003784 | isradipine | CID000004856 | piroxicam | 0.187923 | N |
| CID000003308 | etodolac | CID000004473 | nicardipine | 0.183427 | N |
| CID000002803 | clonidine | CID000004051 | meloxicam | 0.182278 | N |
| CID000004138 | methyldopa | CID000004614 | oxaprozin | 0.177665 | N |
| CID000004051 | meloxicam | CID000004485 | nifedipine | 0.176406 | N |
| CID000003308 | etodolac | CID000004138 | methyldopa | 0.175388 | N |
| CID000003784 | isradipine | CID000004614 | oxaprozin | 0.175243 | N |
| CID000004138 | methyldopa | CID000004856 | piroxicam | 0.173166 | N |
| CID000004138 | methyldopa | CID000005352 | sulindac | 0.165416 | N |
| CID000004473 | nicardipine | CID000004856 | piroxicam | 0.160706 | N |
| CID000003784 | isradipine | CID000005352 | sulindac | 0.150743 | N |
| CID000003519 | guanfacine | CID000005352 | sulindac | 0.149721 | N |
| CID000003519 | guanfacine | CID000004614 | oxaprozin | 0.147719 | N |
| CID000003032 | diclofenac | CID000004499 | nisoldipine | 0.146363 | N |
| CID000003333 | felodipine | CID000004051 | meloxicam | 0.145632 | N |
| CID000004485 | nifedipine | CID000004614 | oxaprozin | 0.14466 | N |
| CID000002244 | aspirin | CID000003519 | guanfacine | 0.144181 | N |
| CID000003075 | diltiazem | CID000084003 | ketorolac tromethamine | 0.143843 | N |
| CID000004138 | methyldopa | CID000084003 | ketorolac tromethamine | 0.143575 | N |
| CID000003519 | guanfacine | CID000003672 | ibuprofen | 0.142196 | N |
| CID000002162 | amlodipine | CID000084003 | ketorolac tromethamine | 0.139603 | N |
| CID000003784 | isradipine | CID000084003 | ketorolac tromethamine | 0.139272 | N |
| CID000003059 | diflunisal | CID000004138 | methyldopa | 0.137675 | N |
| CID000004052 | meloxicam | CID000004499 | nisoldipine | 0.135392 | N |
| CID000002662 | celecoxib | CID000003519 | guanfacine | 0.135213 | N |
| CID000003715 | indomethacin | CID000004473 | nicardipine | 0.132142 | N |
| CID000002520 | verapamil | CID000084003 | ketorolac tromethamine | 0.129593 | N |
| CID000003059 | diflunisal | CID000003075 | diltiazem | 0.128062 | N |
| CID000003059 | diflunisal | CID000003784 | isradipine | 0.128 | N |
| CID000002162 | amlodipine | CID000003059 | diflunisal | 0.125299 | N |
| CID000004499 | nisoldipine | CID000004614 | oxaprozin | 0.122871 | N |
| CID000002803 | clonidine | CID000084003 | ketorolac tromethamine | 0.119673 | N |
| CID000004499 | nisoldipine | CID000005352 | sulindac | 0.119521 | N |
| CID000004473 | nicardipine | CID000005352 | sulindac | 0.117487 | N |
| CID000001302 | naproxen | CID000003519 | guanfacine | 0.116355 | N |
| CID000004499 | nisoldipine | CID000084003 | ketorolac tromethamine | 0.112559 | N |
| CID000003075 | diltiazem | CID000004051 | meloxicam | 0.110873 | N |
| CID000003059 | diflunisal | CID000004499 | nisoldipine | 0.110275 | N |
| CID000004473 | nicardipine | CID000004614 | oxaprozin | 0.109536 | N |
| CID000003672 | ibuprofen | CID000004473 | nicardipine | 0.107915 | N |
| CID000003333 | felodipine | CID000004614 | oxaprozin | 0.1067 | N |
| CID000003519 | guanfacine | CID000004856 | piroxicam | 0.106254 | N |
| CID000004485 | nifedipine | CID000084003 | ketorolac tromethamine | 0.105372 | N |
| CID000002520 | verapamil | CID000003059 | diflunisal | 0.104995 | N |
| CID000003032 | diclofenac | CID000003519 | guanfacine | 0.104932 | N |
| CID000001301 | naproxen | CID000004138 | methyldopa | 0.104913 | N |
| CID000002162 | amlodipine | CID000004051 | meloxicam | 0.102785 | N |
| CID000001301 | naproxen | CID000003784 | isradipine | 0.100114 | N |
| CID000003059 | diflunisal | CID000004473 | nicardipine | 0.098916 | N |
| CID000002803 | clonidine | CID000003059 | diflunisal | 0.09708 | N |
| CID000003059 | diflunisal | CID000004485 | nifedipine | 0.096541 | N |
| CID000004473 | nicardipine | CID000084003 | ketorolac tromethamine | 0.093242 | N |
| CID000001301 | naproxen | CID000004499 | nisoldipine | 0.083435 | N |
| CID000001301 | naproxen | CID000004473 | nicardipine | 0.083064 | N |
| CID000003715 | indomethacin | CID000004499 | nisoldipine | 0.078787 | N |
| CID000002662 | celecoxib | CID000004473 | nicardipine | 0.078377 | N |
| CID000003032 | diclofenac | CID000004473 | nicardipine | 0.077065 | N |
| CID000001302 | naproxen | CID000004473 | nicardipine | 0.072841 | N |
| CID000001301 | naproxen | CID000002162 | amlodipine | 0.068179 | N |
| CID000003519 | guanfacine | CID000004052 | meloxicam | 0.067737 | N |
| CID000003308 | etodolac | CID000004499 | nisoldipine | 0.067679 | N |
| CID000001301 | naproxen | CID000003075 | diltiazem | 0.065316 | N |
| CID000004051 | meloxicam | CID000004138 | methyldopa | 0.059949 | N |
| CID000003784 | isradipine | CID000004051 | meloxicam | 0.056104 | N |
| CID000003333 | felodipine | CID000084003 | ketorolac tromethamine | 0.047876 | N |
| CID000004051 | meloxicam | CID000004473 | nicardipine | 0.047619 | N |
| CID000004051 | meloxicam | CID000004499 | nisoldipine | 0.046171 | N |
| CID000003059 | diflunisal | CID000003519 | guanfacine | 0.037152 | N |
| CID000001301 | naproxen | CID000002520 | verapamil | 0.031369 | N |
| CID000003784 | isradipine | CID000004052 | meloxicam | 0.026207 | N |
| CID000001301 | naproxen | CID000002803 | clonidine | 0.025687 | N |
| CID000003059 | diflunisal | CID000003333 | felodipine | 0.025453 | N |
| CID000001301 | naproxen | CID000004485 | nifedipine | 0.01907 | N |
| CID000004499 | nisoldipine | CID000004856 | piroxicam | 0.016996 | N |
| CID000001301 | naproxen | CID000003519 | guanfacine | 0 | N |
| CID000003519 | guanfacine | CID000004051 | meloxicam | 0 | N |
| CID000003519 | guanfacine | CID000084003 | ketorolac tromethamine | 0 | N |

**Table S3 - Prediction scores between cholesterol-lowering statin drugs and antibiotics clarithromycin/erythromycin**

In the table, statin drugs include atorvastatin, fluvastatin, lovastatin, pravastatin, simvastatin, rosuvastatin calcium, and rosuvastatin.

| DRUG1_ID | DRUG1_NAME | DRUG2_ID | DRUG2_NAME | PRED_SCORE | PRED_LABEL |
| --- | --- | --- | --- | --- | --- |
| CID000003403 | fluvastatin | CID000054688 | clarithromycin | 0.420365 | Y |
| CID000054688 | clarithromycin | CID000446157 | rosuvastatin calcium | 0.393997 | Y |
| CID000003255 | erythromycin | CID000446157 | rosuvastatin calcium | 0.335658 | Y |
| CID000003403 | fluvastatin | CID000071469 | erythromycin lactobionate | 0.321351 | Y |
| CID000071469 | erythromycin lactobionate | CID004475485 | rosuvastatin | 0.258013 | Y |
| CID000071469 | erythromycin lactobionate | CID000446157 | rosuvastatin calcium | 0.257837 | Y |
| CID000003962 | lovastatin | CID000071469 | erythromycin lactobionate | 0.227563 | Y |
| CID000054454 | simvastatin | CID000071469 | erythromycin lactobionate | 0.226516 | Y |
| CID000004889 | pravastatin | CID000071469 | erythromycin lactobionate | 0.207577 | Y |
| CID000002250 | atorvastatin | CID000071469 | erythromycin lactobionate | 0.184649 | N |

**Table S4 - Prediction scores between Selective Serotonin Reuptake Inhibitor (SSRI) antidepressants and hydrocodone**

In the table, SSRI antidepressants include citalopram, fluoxetine, fluvoxamine, paroxetine, and sertraline.

| DRUG1_ID | DRUG1_NAME | DRUG2_ID | DRUG2_NAME | PRED_SCORE | PRED_LABEL |
| --- | --- | --- | --- | --- | --- |
| CID000002771 | citalopram | CID000411697 | hydrocodone | 0.503992 | Y |
| CID000003386 | fluoxetine | CID000411697 | hydrocodone | 0.37671 | Y |
| CID000003404 | fluvoxamine | CID000411697 | hydrocodone | 0.264713 | Y |
| CID000004691 | paroxetine | CID000411697 | hydrocodone | 0.476755 | Y |
| CID000005203 | sertraline | CID000411697 | hydrocodone | 0.472999 | Y |

**Section 2: Analysis of Tanimoto coefficient (TC) to compute similarity between drug SE profiles**

We used Tanimoto coefficient (TC) to compute similarity between drug SE profiles. The TC between two SE profiles A and B is defined as the ratio between the number of SE terms in the intersection to the union of both profiles: TC(A,B) = |A∩B|/|A∪B|. Let α＝|A∩B|, β=|A∪B|, Δ denotes the number of SE terms which are shared by both drugs (A and B) and can be combined into other SE terms. Then the difference between the TC of original SE profiles (TC_original_) and the TC after removing all redundant SE terms (i.e., combining all synonyms into one SE term, TC_after_) is:

$$TC_{original}-TC_{after}=\frac{\alpha}{\beta}-\frac{\alpha-\Delta}{\beta-\Delta}=\frac{(\beta-\alpha)\Delta}{\beta(\beta-\Delta)}=(\frac{\beta}{\beta-\Delta}-1)(1-\frac{\alpha}{\beta})$$

Because β is usually much larger than Δ (the mean of β is 179.45 in our data, Δ is 0 or a very small positive number - as the chance of two drugs share a group of very similar SE terms is rare), the first term approaches to 0, resulting in the difference between TC_original_ and TC_after_ approaches to 0. Therefore, TC is not sensitive to the dimensionality, and the multiple-name problem doesn’t have a significant impact on the SE similarity measurement.
